# Student's Learning Strategies and Academic Emotions: Their Influence on Learning Satisfaction During the COVID-19 Pandemic

**DOI:** 10.3389/fpsyg.2021.717683

**Published:** 2021-09-24

**Authors:** Changcheng Wu, Bin Jing, Xue Gong, Ya Mou, Junyi Li

**Affiliations:** ^1^School of Computer Science, Sichuan Normal University, Chengdu, China; ^2^Faculty of Artificial Intelligence in Education, Central China Normal University, Wuhan, China; ^3^Shuang Liu Middle School, Chengdu, China; ^4^School of Psychology, Sichuan Normal University, Chengdu, China

**Keywords:** online learning, learning satisfaction, academic emotion, learning strategy, behavioral engagement, social interaction

## Abstract

**Background:** Based on the control-value theory (CVT), learning strategies and academic emotions are closely related to learning achievement, and have been considered as important factors influencing student's learning satisfaction and learning performance in the online learning context. However, only a few studies have focused on the influence of learning strategies on academic emotions and the interaction of learning strategies with behavioral engagement and social interaction on learning satisfaction.

**Methods:** The participants were 363 pre-service teachers in China, and we used structural equation modeling (SEM) to analyze the mediating and moderating effects of the data.

**Results:** The main findings of the current study showed that learning strategies influence students' online learning satisfaction through academic emotions. The interaction between learning strategies and behavioral engagement was also an important factor influencing online learning satisfaction.

**Conclusions:** We explored the internal mechanism and boundary conditions of how learning strategies influenced learning satisfaction to provide intellectual guarantee and theoretical support for the online teaching design and online learning platform. This study provides theoretical contributions to the CVT and practical value for massive open online courses (MOOCs), flipped classrooms and blended learning in the future.

## Introduction

During the first half of 2020, the world faced the unprecedented COVID-19 pandemic. This crisis has presented new challenges and opportunities to the global educational system, and these features forced teachers and students to quickly migrate their courses online to prevent the spread of the virus that causes COVID-19 (Hodges et al., 2020). Some researchers have believed that online instruction can lead to the flexibility of teaching at any time and any place. This practice provides a rare opportunity for the development of online learning. However, other studies have shown that the quality of online teaching during the crisis is not high, indicating that this type of learning can only be used as a special temporary measure, and face-to-face learning should be returned as soon as possible after the crisis (Hodges et al., 2020).

This paradigm is being promoted because during the crisis, most teachers and students are forced to switch to online learning. These stakeholders are not accustomed to this learning setup, leading to low learning satisfaction and even high dropout rates (Dai et al., 2020). Baber (2020) and Chao (2019) indicated that improving learning satisfaction is important to increase the student's intention to continue online learning and reduce dropout rates. Although online learning platform developers have invested a huge amount of money and several types of new technologies, students are not as satisfied as expected (Jiang et al., 2021). In previous studies, the evaluation index of online teaching quality is mainly cognitive learning performance, ignoring learning satisfaction on the psychological level (Méndez-Aguado et al., 2020). Therefore, in this study, we focused on some of the learning process variables to address the forementioned defects. These variables had been proven to influence learning satisfaction and performance. On the one hand, given that academic emotions and the use of learning strategies are important for successful learning, more and more studies have investigated the relationship between these variables (Ahmed et al., 2013; Mariza et al., 2015; de la Fuente et al., 2020; Obergriesser and Stoeger, 2020). However, the exact relationship between students' academic emotions and their use of learning strategies has not been fully understood (Obergriesser and Stoeger, 2020). Most previous studies have explored the influence of academic emotions on learning strategies and achievements (Ahmed et al., 2013; Mariza et al., 2015). A recent study has explored the influence of learning strategies on academic emotions (de la Fuente et al., 2020; Obergriesser and Stoeger, 2020). Thus, we focused on how these two variables influence learning satisfaction. On the other hand, behavioral engagement and social interaction had also been proved to be lacking in online learning (Muilenburg and Berge, 2005; Guo et al., 2019) and to promote learning satisfaction (Gray and DiLoreto, 2016; Nagy, 2018). However, the manner by which they influence learning satisfaction has not been determined yet. In addition, migrating to online instruction requires teachers to clarify the characteristics of learning under the state of separation of time and space and perform corresponding online instructional designs to arrange relevant learning activities (Karakaya, 2021). Hence, such activity necessitates teachers to control more the course design, development, and implementation (Karakaya, 2021). Effective online learning is the result of meticulous planning and instructional design. However, most teachers have not performed well enough at this stage.

Online learning during the COVID-19 pandemic is only a temporary alternative due to the crisis. In an emergency, institutions should not only ensure the safety of teachers and students but also the quality of teaching (Hodges et al., 2020). This situation has put forward higher requirements for future online learning, and instruction should return to educational rationality and may consider providing better support to students (Karakaya, 2021). Therefore, the overall aim of this study is to examine the internal mechanism and boundary conditions on the relationship between learning strategies and satisfaction. This study also provides intellectual guarantee and theoretical support for online learning in the post-pandemic era.

## Theoretical Framework

### Learning Satisfaction

Learning satisfaction can be defined as the student's perception of the course or learning experience and of the value of receiving education in an educational institution (Ke and Kwak, 2013). Considering the concerns on the dropout rates (Hew et al., 2020), this study follows the advice of Rabin et al. (2019) to use learning satisfaction as one of the important indicators to measure the success of online learning. Learning satisfaction is also an indispensable result for students because it influences the student's motivation, which is an important psychological factor that influences learning (Hew et al., 2020). In recent years, academic circles have paid increasing attention to the study of learning satisfaction and its potential determinants. According to the *Sloan Consortium*, an American non-profit organization dedicated to improving the quality of online learning, learning satisfaction is one of the most important factors in evaluating the quality of online learning (Moore, 2005). Some studies have also shown that learning satisfaction is a key factor for students to decide whether to continue to choose the course and is a significant predictor of learning grades to improve the sustainability of online learning (Ke and Kwak, 2013; Chao, 2019).

### Learning Strategies

Learning strategies are processes to obtain, organize, or transform information (Alexander et al., 1998). In this study, we divided learning strategies into three categories, namely, metacognitive self-regulation, elaboration, and organization strategies. In particular, students use metacognitive self-regulation strategies to mobilize various consciousness and behavior to participate in the learning process, which can help students effectively implement cognitive strategies (Obergriesser and Stoeger, 2020). Using elaboration strategies to establish connections between new materials and visual imagination or semantic knowledge can increase the meaning of new information (Wolters et al., 2005). Organization strategies for establishing relationships between different parts of learning material can help students select and organize information and create meaningful units of information (Obergriesser and Stoeger, 2020). Studies have shown that the student's use of these strategies has a significant and positive correlation with learning satisfaction (Choi, 2016; Kasalak and Dagyar, 2020). Students who flexibly use learning strategies have also been shown to better perceive the control of the learning process (Obergriesser and Stoeger, 2020). This phenomenon influences the student's self-efficacy, academic emotions, and learning outcome (Pekrun et al., 2011; Murayama et al., 2013; Pekrun and Perry, 2014). These conclusions are supported by the affective dynamics model of D'Mello and Graesser (2012). This model assumes that during learning, by effectively using learning strategies to eliminate learning obstacles, negative emotions will be reduced, and students will enjoy their learning. That is, learning strategies can promote the enhancement of student's positive emotions and reduce their negative emotions (Muis et al., 2015a). However, some studies have shown that although the application of learning strategies has no significant influence on the student's positive emotions, it can significantly reduce negative emotions (Obergriesser and Stoeger, 2020). Previous studies mainly focused on the influence of academic emotions on learning strategies (Ahmed et al., 2013; Mariza et al., 2015), while few studies have explored the influence of learning strategies on academic emotions and their two-way influence. Hence, no consensus on how the learning strategies influence the student's academic emotions during online learning is available.

### Academic Emotions

Academic emotions are directly related to the learning process and results. A relatively stable and long-term emotional state and a complex subjective experience of students are involved through the whole learning process (Pekrun, 2006). According to valence, academic emotions can be divided into positive and negative emotions (Pekrun et al., 2011). In this study, we focused on activity-related emotions, namely, enjoyment (i.e., positive emotions), boredom, and frustration (i.e., negative emotions) (Pekrun et al., 2011). Studies have shown that academic emotions will influence learning satisfaction (Artino and Jones, 2012; Lee et al., 2021). Moreover, under the traditional face-to-face teaching model, academic emotions are important predictors of the learning process and results (Pekrun et al., 2009). Positive emotions can stimulate intrinsic and extrinsic motivation and have a positive influence on academic performance in most cases. Negative emotions have the opposite effects (Pekrun et al., 2011). Sewart (1993) showed that if teachers and online learning platforms fail to timely detect the emotional changes of students and provide relevant emotional support, students will easily suffer from negative emotions, such as boredom and frustration, gradually lose interest in learning, and even drop out from learning. However, some studies have found that negative emotions have positive effects on the use of metacognitive self-regulation strategies and the development of online group activities during online learning (Artino and Jones, 2012; Noteborn et al., 2012). Hence, the mechanism by which the student's academic emotions influence their learning satisfaction needs further study.

### Behavioral Engagement

In recent decades, learning engagement has been paid increasing attention. Learning engagement is depicted as a “meta” construct, consisting of three dimensions, namely, behavioral, cognitive, and emotional engagement (Luo et al., 2019). In this study, we focused on behavioral engagement, which is described as the time, effort, attention, and perseverance of students to complete tasks. Behavioral engagement is positively related to emotional engagement, such as learning interest or satisfaction (Fredricks et al., 2004). Previous studies have shown that behavioral engagement is closely related to learning satisfaction (Gray and DiLoreto, 2016; Luo et al., 2019). If students can be fully engaged in learning activities, their learning satisfaction will be correspondingly improved. With the rapid development of information technology, the study on behavioral engagement has transformed from focusing on the individual behavior and efforts of students to an important factor to improve the quality of online education.

### Social Interaction

In learning activities, social interaction is also an essential link. The process of interaction is not only the process of learning but also of improving social skills. Moore (1989) proposed that social interaction includes the interaction between students and teachers, students and students, and students and learning content. Studies have shown that the interaction between students has a stronger influence on online learning satisfaction than that between students and teachers (Jung et al., 2002; Nagy, 2018). In online learning, students interact with other students through various communication channels, such as danmakus, comments, and replies. In the current study, we focused on danmakus, a kind of behavior of students sending messages on the screen. As a new type of social interaction, this behavior is widely favored by teenagers, especially for providing real-time communication for students. This behavior can stimulate their interest in learning and improve their learning satisfaction (Leng et al., 2016; Wu et al., 2019). However, the latest studies have shown that during video teaching, danmakus can both promote and hinder learning satisfaction (Yang et al., 2019). Danmakus related to learning content may improve learning satisfaction, which may be reduced by unrelated danmakus (Zhang et al., 2019).

### The Control-Value Theory

**Figure 1:**
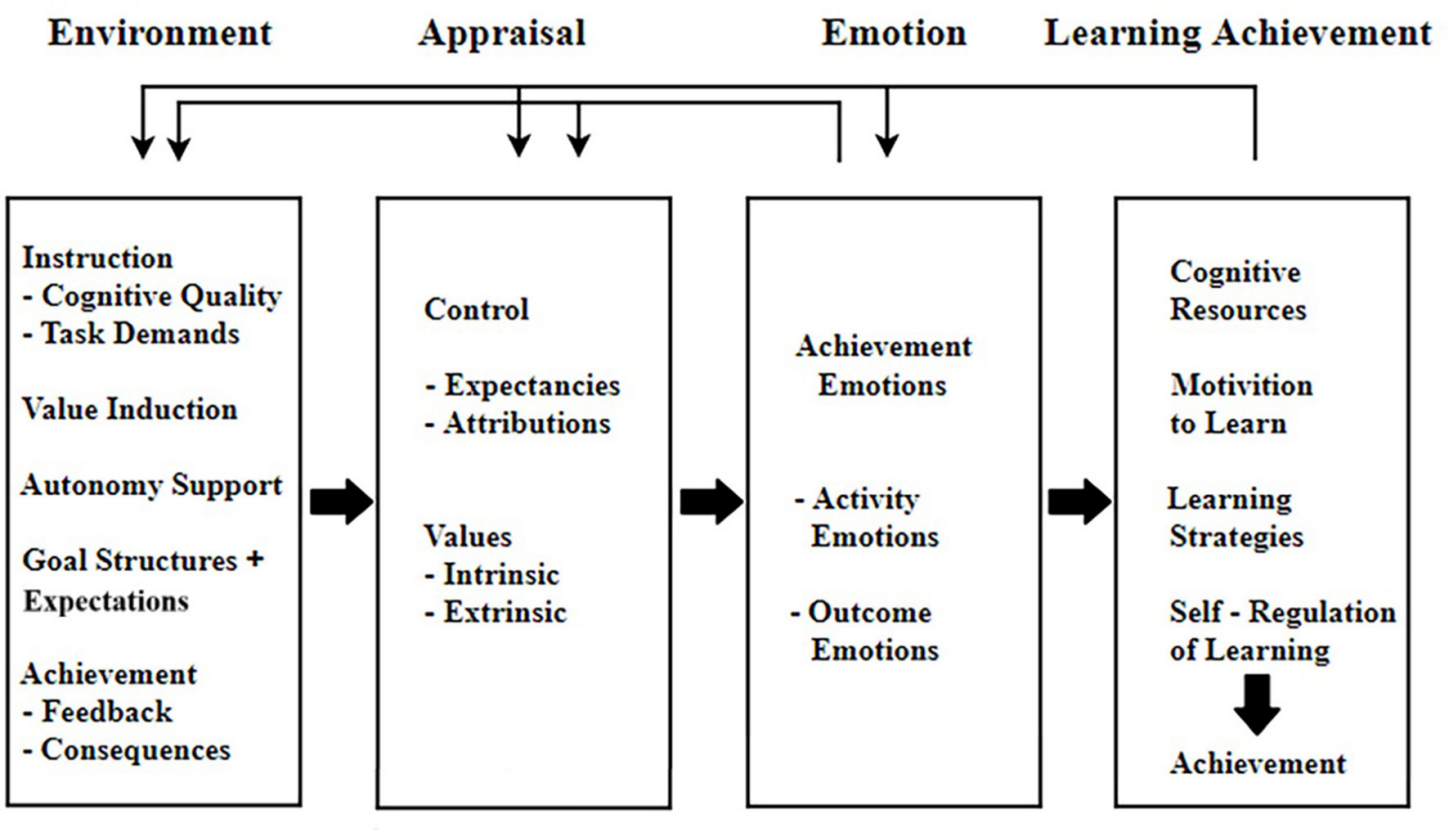
The control-value theory (partial) (Pekrun, 2006).

Given that control and value appraisals can be postulated to be precursors of emotions, Pekrun (2006) proposed the CVT to analyze the causes and consequences that influence academic emotions (Figure 1).

The CVT is used to analyze the relationship among academic emotions, learning environment, and cognitive evaluation factors. This theory provides a theoretical basis for the methodology of academic emotions (Pekrun, 2006). Control refers to the student's perception and judgment of the controllability of the learning process and results. Value describes the value that students give to the learning task or results, such as how important the learning task is to themselves (Pekrun, 2006). Theoretically, a dynamic cycle of interaction exists among environment, appraisal, emotion, and learning achievement; that is, the influence between academic emotions and learning strategies is a two-way relationship (Pekrun, 2006; Pekrun and Perry, 2014). Learning strategies are also some of the important factors influencing academic emotions, which will further influence the learning process and results.

### Hypotheses

Hence, learning strategies, academic emotions, behavioral engagement, and social interaction are the core variables that influence learning satisfaction. Learning strategies and academic emotions can influence learning satisfaction, and this satisfaction can also be influenced by behavioral engagement and social interaction. Moreover, according to the CVT (Pekrun, 2006), learning strategies may influence learning satisfaction by influencing academic emotions. However, few studies have explored the mechanism by which learning strategies indirectly influence learning satisfaction by influence learning emotions. In addition, behavioral engagement and social interaction are used as boundary conditions for learning strategies to influence learning satisfaction. Although insights into the relationships between constructs have been provided, studies do not make causal inferences. Therefore, mediating and moderating models were constructed in the current study. We address the following hypotheses:

H1: Learning strategies positively predict positive emotions and negatively predict negative emotions.
H2: Positive emotions positively predict learning satisfaction, while negative emotions negatively predict learning satisfaction.
H3: Academic emotions have a mediating effect on the relationship between learning strategies and learning satisfaction.
H4: Behavioral engagement and social interaction have a moderating effect on the relationship between learning strategies and learning satisfaction, respectively.

## Method

### Context

During the COVID-19 pandemic, to reduce the spread of the virus and protect the health and safety of teachers and students, educational institutions in various countries canceled all face-to-face learning activities and encouraged teachers and students to conduct online teaching. As a result, tens of millions of students suddenly switched from traditional face-to-face learning to fully online learning. Therefore, in the spring semester of 2020, a university in Chengdu, Sichuan, China, was investigated by class as a unit. Taking the online course “*Computer Science Fundamentals*” as an example, the rain classroom (an intelligent online teaching platform) was adopted for teaching. The relevant empirical study was conducted on learning satisfaction and its influencing factors.

### Participants

In this study, 363 pre-service teachers (*M*_age_ = 18.95, *SD*_age_ = 0.88) who participated in online learning were the participants (Table 1). They completed an online survey (www.wjx.cn) in ~15 min voluntarily and anonymously.

**Table 1:** The basic information of participants.

**Characteristic**	**Variable**	**Quantity**	**Proportion**
Gender	Male	128	35.26%
	Female	235	64.74%
Household	Country	69	19.01%
	Town	90	24.79%
	City	204	56.20%
Major	Accountancy	58	15.98%
	Dance	10	2.75%
	Music performance	98	27.00%
	Apparel fashion design	164	45.18%
	Social sports guidance and management	33	9.09%

### Measurements

#### Learning Strategies

The learning strategy questionnaires developed by Pintrich and De Groot (1990) and Duncan and McKeachie (2005) were used to revise it. The questionnaire was translated into Chinese and developed by Kong and Lu (2012) and Xiong et al. (2012). This study consisted of 22 items with a five-point Likert scale (from 1 = strongly disagree to 5 = strongly agree), which was divided into three dimensions of metacognitive self-regulation (12 items, e.g., “I ask myself questions to make sure I understand the material I have been studying in this class”), elaboration (six items, e.g., “When reading for this class, I try to relate the material to what I already know”), and organization strategies (four items, e.g., “When I study for this course, I go over my class notes and make an outline of important concepts”), to measure the student's use of learning strategies during online learning. The overall internal consistency α coefficient of this Chinese questionnaire was 0.96 (the corresponding α values for the three dimensions were 0.90, 0.96, and 0.95), which indicated the high reliability quality.

#### Academic Emotions

This questionnaire was adapted from the Achievement Emotion Questionnaire (AEQ) developed by Pekrun et al. (2011). The questionnaire was translated into Chinese and developed by Gong et al. (2016). The survey had three dimensions, and a total of 13 items with a five-point Likert scale (from 1 = strongly disagree to 5 = strongly agree), namely, enjoyment (4 items, e.g., “I am enjoying the online course”), boredom (five items, e.g., “I feel bored while studying the online course”), and frustration (four items, e.g., “I felt very frustrated when studying the online course”), to measure the student's academic emotions during online learning (Artino and Jones, 2012). The overall internal consistency α coefficient was 0.85 (the corresponding α values of the three dimensions were 0.91, 0.96, and 0.96), which indicated the high reliability of this Chinese questionnaire.

#### Learning Satisfaction

This questionnaire was derived from the Chinese-language Video Course Learning Satisfaction Questionnaire (Yang, 2014). According to the actual needs, some items were appropriately deleted and modified in this study to adapt to the online teaching context, and 10 items (e.g., “Overall, I was satisfied with the teaching of this online course.”) were retained at last. A five-point Likert scale (from 1 = strongly disagree to 5 = strongly agree) was used for the questionnaire options. The purpose of this questionnaire was to measure the learning satisfaction of students for online courses. The internal consistency α coefficient was 0.93 in this study, which indicated the high reliability of the questionnaire.

#### Behavioral Engagement

The course teaching in this study was based on the rain classroom. Students read online courseware related to the course content, the number of check-in times in class, and the number of times of reading the bulletin board, which were defined as behavioral engagement. Data were automatically generated and exported through the backstage of this platform. They were divided into low and high behavioral engagement groups according to the median. Behavioral engagement was coded 1 = low group, 2 = high group.

#### Social Interaction

Social interaction mainly referred to the student's interactive behavior of sending danmakus, specifically referring to the number of danmakus messages sent. Social interaction data were also automatically recorded, generated, and exported backstage of the rain classroom. The data were divided into low and high social interaction groups according to the median. Social interaction was coded 1 = low group, 2 = high group.

### Data Collection and Analysis

Data were collected in May 2020. First, the questionnaire was uploaded to WJX (www.wjx.cn). Then, the data were entered and managed using Excel 2019, and SPSS 24.0 was used for descriptive statistical analysis and correlation analysis. Finally, the mediating and moderating effects of the model were analyzed in Mplus 8.3 by SEM analysis. The deviation-corrected percentile Bootstrapping method was used to test (repeat sampling, 2,000 times).

## Results

### Descriptive Statistics and Correlation Analysis

In this study, the mean, standard deviation, and Pearson correlation coefficient of the learning strategies (i.e., metacognitive self-regulation, elaboration, and organization strategies), academic emotions (i.e., positive emotions and negative emotions), behavioral engagement, social interaction, and learning satisfaction were analyzed (Table 2).

**Table 2:** Descriptive statistics and correlation analysis of each variable.

**Variable**	**M**	**SD**	**1**	**2**	**3**	**4**	**5**	**6**	**7**	**8**
1. Metacognitive self-regulation strategies	3.68	0.62	—							
2. Elaboration strategies	3.94	0.74	0.70^**^	—						
3. Organization strategies	3.74	0.79	0.80^**^	0.71^**^	—					
4. Positive emotions	3.80	0.89	0.44^**^	0.45^**^	0.40^**^	—				
5. Negative emotions	2.01	0.95	−0.40^**^	−0.35^**^	−0.34^**^	−0.31^**^	—			
6. Behavioral engagement	13.79	3.68	0.14^**^	0.08	0.02	0.06	−0.17^**^	—		
7. Social influence	22.51	31.63	−0.04	−0.09	−0.13^*^	−0.02	−0.02	0.23^**^	—	
8. Gender	1.65	0.48	0.10	−0.05	−0.04	0.01	−0.10	0.25^**^	−0.02	—
9. Learning Satisfaction	4,30	0.60	0.59^**^	0.65^**^	0.59^**^	0.49^**^	−0.46^**^	0.14^**^	−0.02	0.05

*M, mean; SD, standard deviation*;

* *p < 0.05*,

** *p < 0.01*.

The results showed that learning strategies were positively linked with positive emotions (*r* = 0.44, *p* < 0.01; *r* = 0.45, *p* < 0.01; *r* = 0.40, *p* < 0.01) and learning satisfaction (*r* = 0.59, *p* < 0.01; *r* = 0.65, *p* < 0.01; *r* = 0.59, *p* < 0.01) but negatively linked with negative emotions (*r* = −0.40, *p* < 0.01; *r* = −0.35, *p* < 0.01; *r* = −0.34, *p* < 0.01). Positive emotions were positively linked with learning satisfaction (*r* = 0.49, *p* < 0.01) and were negatively linked with negative emotions (*r* = −0.31, *p* < 0.01). Negative emotions were negatively linked with learning satisfaction (*r* = −0.46, *p* < 0.01). Metacognitive self-regulation strategies were positively linked with behavioral engagement (*r* = 0.14, *p* < 0.01). Organization strategies were negatively linked with social interaction (*r* = 0.13, *p* < 0.05). The behavioral engagement was positively linked with social interaction (*r* = 0.23, *p* < 0.01), gender (*r* = 0.25, *p* < 0.01), and learning satisfaction (*r* = 0.14, *p* < 0.01).

### Analysis of the Mediating Effect of Academic Emotions

**Figure 2:**
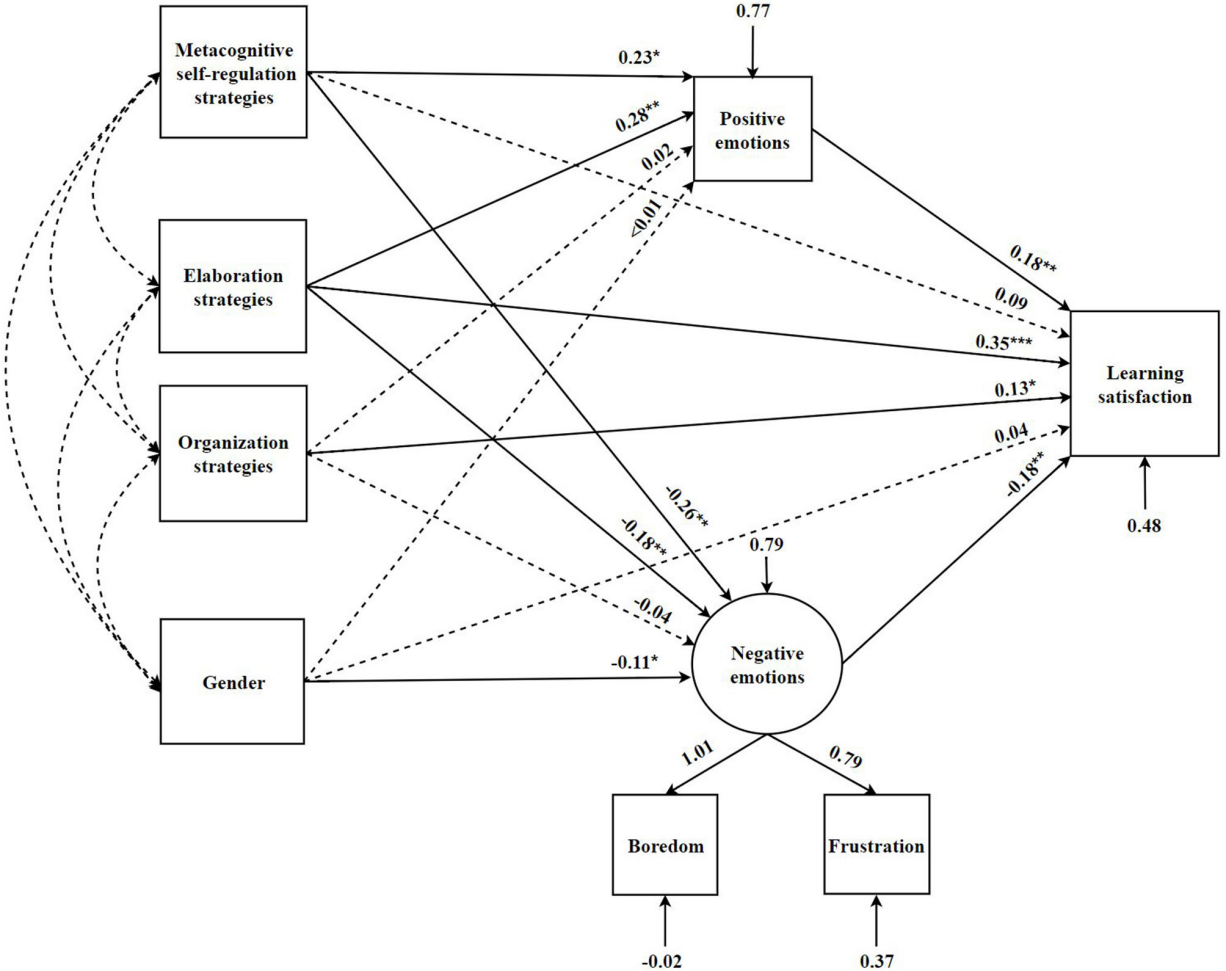
The influence of learning strategies on learning satisfaction: a mediating model of academic emotions. *p < 0.05, **p < 0.01, ***p < 0.001.

In this study, learning strategies were considered as the independent variables, academic emotions were the mediating variables, and learning satisfaction was the dependent variable to build a mediating model (Figure 2). This model used the χ^2^ value by the degrees of freedom (χ^2^*/df*), root mean square error of approximation (*RMSEA*), comparative fit index (*CFI*), and Tucker-Lewis index (*TLI*) to evaluate the fit index in the current study. The results showed that the model fit well (χ^2^ = 16.37, *df* = 6, χ^2^*/df* = 2.73, *p* < 0.05; *RMSEA* = 0.07, *CFI* = 0.99, *TLI* = 0.95). The significance of the mediating effect was analyzed by the bias correction Bootstrapping method (Table 3). If the 95% confidence interval of the average estimate of the mediating effect of a path in the model did not contain 0, the mediating effect corresponding to the path was significant (Shrout and Bolger, 2002).

**Table 3:** Path coefficients of the initial structural model.

**Variable**		**β**	***se***	***t***	***p***
Metacognitive self-regulation strategies	Positive emotions	0.23^*^	0.09	2.45	<0.05
	Negative emotions	−0.26^**^	0.09	−2.94	<0.01
	Learning satisfaction	0.09	0.07	1.34	>0.05
Elaboration strategies	Positive emotions	0.28^**^	0.08	3.45	<0.01
	Negative emotions	−0.18^**^	0.07	−2.77	<0.01
	Learning satisfaction	0.35^***^	0.06	6.09	<0.001
Organization strategies	Positive emotions	0.02	0.08	0.24	>0.05
	Negative emotions	−0.04	0.08	−0.51	>0.05
	Learning satisfaction	0.13^*^	0.06	2.12	<0.05
Gender	Positive emotions	<0.01	0.05	0.07	>0.05
	Negative emotions	−0.11^*^	0.05	−2.11	<0.05
	Learning satisfaction	0.04	0.04	1.00	>0.05
Positive emotions	Learning satisfaction	0.18^**^	0.05	3.38	<0.01
Negative emotions		−0.18^**^	0.05	−3.33	<0.01

*β, standardized path coefficient; se, standard error; t, t-statistic*;

* *p < 0.05*,

** *p < 0.01*,

*** *p < 0.001*.

Table 3 demonstrated the hypotheses testing results for direct path coefficients of initial structural model. In the condition of gender being controlled, the results showed that metacognitive self-regulation self-regulation strategies had significant influence on positive emotions (β = 0.23, *t* = 2.45, *p* < 0.05) and negative emotions (β = −0.26, *t* = −2.94, *p* < 0.01), whereas its predictive effect on learning satisfaction was not significant. Additionally, positive emotions (β = 0.28, *t* = 3.45, *p* < 0.01), negative emotions (β = −0.18, *t* = −2.77, *p* < 0.01), and learning satisfaction (β = 0.35, *t* = 6.09, *p* < 0.001) were significantly predicted by elaboration strategies. As for organization strategies, we only found a significant direct effect between organization strategies and learning satisfaction (β = 0.13, *t* = 2.12, *p* < 0.05). Furthermore, learning satisfaction was significantly predicted by positive emotions (β = 0.18, *t* = 3.38, *p* < 0.01), and negative emotions (β = −0.18, *t* = −3.33, *p* < 0.01). Finally, regarding gender, we did not find any effect of gender on positive emotions or learning satisfaction, but gender significantly predicted negative emotions (β = −0.11, *t* = −2.11, *p* < 0.05).

The direct and mediating effects of learning strategies on learning satisfaction were examined using the Bootstrapping method (Table 4). First, elaboration strategies (*g* = 0.35, *p* < 0.001) and organization strategies (*g* = 0.13, *p* < 0.05) had direct influence on learning satisfaction, while metacognitive self-regulation strategies had no direct influence on learning satisfaction. Second, positive emotions (*g* = 0.04, *p* < 0.05) and negative emotions (*g* = 0.05, *p* < 0.05) had fully mediating effects on the relationship between metacognitive self-regulation strategies and learning satisfaction, respectively. Positive emotions (*g* = 0.05, *p* < 0.05) and negative emotions (*g* = 0.03, *p* < 0.05) partially mediated the relationship between elaboration strategies and learning satisfaction. Finally, positive emotions did not show a mediating effect on the relationship between organization strategies and learning satisfaction, nor did negative emotions.

**Table 4:** Bootstrapping analysis of the mediating effect test.

**Dependent variable**	**Independent variable**	**Mediating variable**	**Direct effect**	**Mediating effect**	**LLCI**	**ULCI**
Learning satisfaction	Metacognitive self-regulation strategies	Positive emotions	0.09	0.04^*^	0.01	0.09
		Negative emotions	0.09	0.05^*^	0.01	0.10
	Elaboration strategies	Positive emotions	0.35^***^	0.05^*^	0.02	0.11
		Negative emotions	0.35^***^	0.03^*^	0.01	0.07
	Organization strategies	Positive emotions	0.13^*^	—	−0.02	0.04
		Negative emotions	0.13^*^	—	−0.02	0.04

*LLCI, lower level of confidence interval; ULCI, upper level of confidence interval*;

* *p < 0.05*,

*** *p < 0.001*.

The results showed that the student's metacognitive self-regulation strategies indirectly influenced learning satisfaction by influencing their academic emotions. Elaboration strategies not only directly influenced learning satisfaction but also indirectly influenced learning satisfaction by influencing academic emotions. Although we found that organization strategies directly inflence learning satisfaction. However, organization strategies did not indirectly influence learning satisfaction through academic emotions. Specifically, if students often used metacognitive self-regulation and elaboration strategies, they experienced more positive emotions and less negative emotions during the learning process and thus had higher learning satisfaction. In addition, male students reported more negative emotions than female students.

### Analysis of Moderating Effect of Behavioral Engagement and Social Interaction

**Figure 3:**
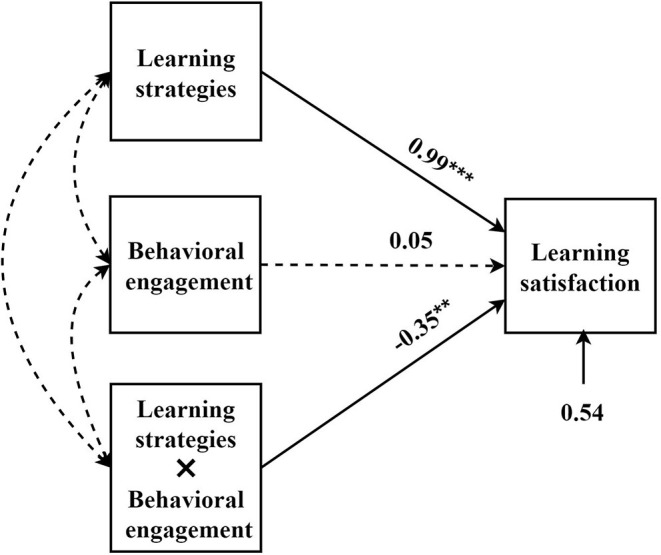
The influence of learning strategies on learning satisfaction: a moderating model of behavioral engagement. **p < 0.01, ***p < 0.001.

**Figure 4:**
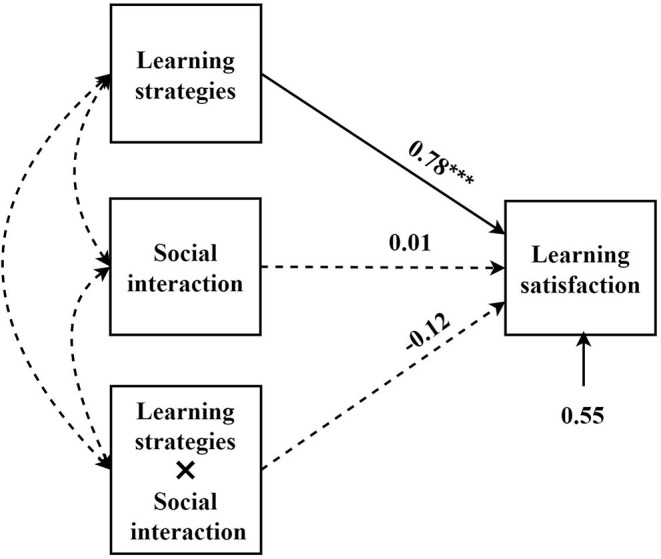
The influence of learning strategies on learning satisfaction: a moderating model of social interaction. ***p < 0.001.

In this study, learning strategies were used as the independent variables, learning satisfaction as the dependent variable, and behavioral engagement and social interaction were the moderating variables to build the moderating model (Figures 3, 4).

**Figure 5:**
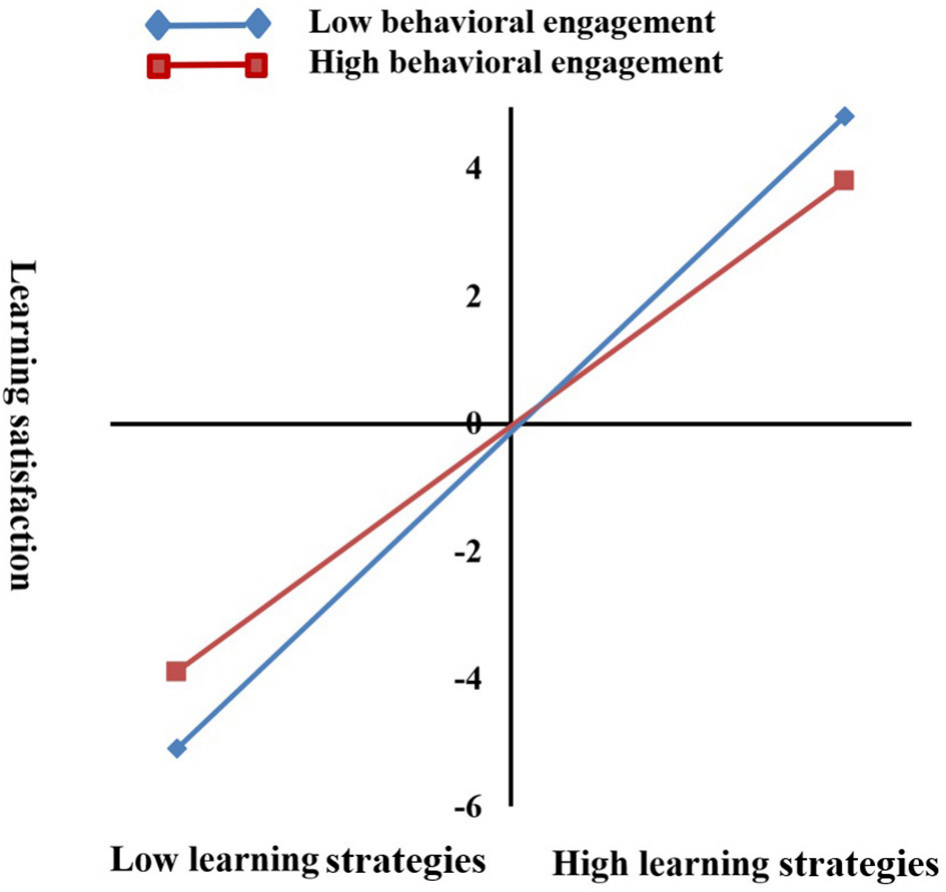
The influence of learning strategies on learning satisfaction: a simple slope figure of the moderating effect.

From the moderating models that we constructed, behavioral engagement had a moderating effect on the relationship between learning strategies and learning satisfaction, while social interaction had no moderating effect. From the simple slope figure of moderating effect (Figure 5), under low-level learning strategies, a high level of behavioral engagement increased learning satisfaction, and low-level behavioral engagement reduced the learning satisfaction. By contrast, under the high-level learning strategy, the scenario was the opposite.

The results showed that the student's behavioral engagement had a moderating effect on the relationship between learning strategies and satisfaction. With the increase in the student's use of learning strategies, excessive behavioral engagement would negatively reduce learning satisfaction. Nonetheless, the moderating effect of social interaction on the relationship between learning strategies and satisfaction was not significant.

**Figure 6:**
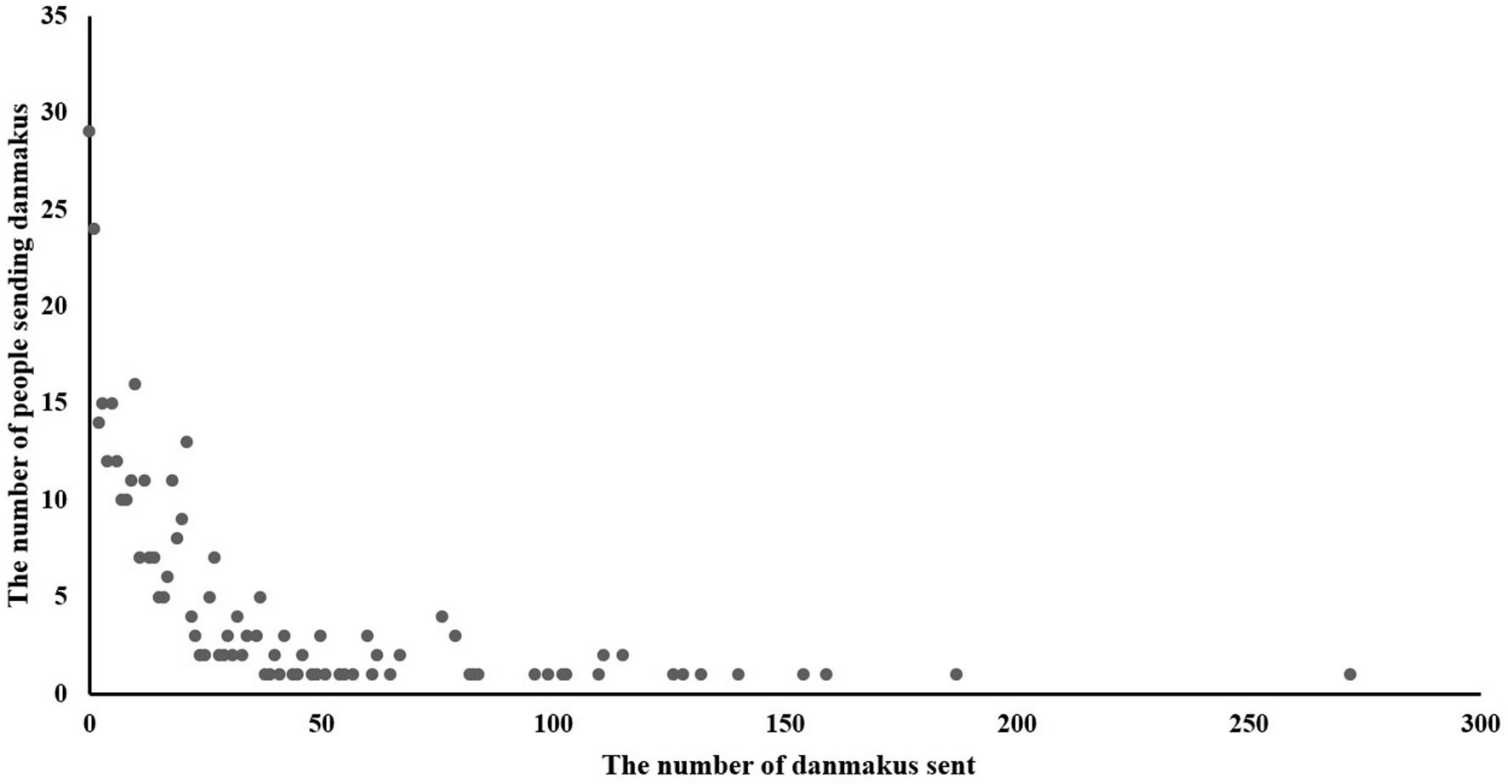
The scatter figure of the number of people sending danmakus and the number of danmakus sent.

Figure 6 shows that during online learning, the student's social interaction presented a polarization trend. Most students did not send danmakus or rarely sent them. Only a minority of students liked to send a large number of danmakus.

## Discussion

Due to the COVID-19 pandemic, the vast majority of college students around the world rely on online learning platforms to continue their studies. This massive online learning activity will facilitate the sustainable development of blended learning in the post-pandemic era. Some studies have suggested that learning satisfaction is correlated with a stronger intention to participate in online learning (Salam and Farooq, 2020), lower dropout rates (Hew et al., 2020), and better learning performance (Al-Fraihat et al., 2020). Therefore, measuring the learning satisfaction of students on online learning platforms is urgent. Hence, the purpose of the current study is to respond to this need by examining how learning strategies predict academic emotions and how do these variables predict learning satisfaction. In addition, the manner by which the interaction of learning strategies with behavioral engagement and social interaction may predict learning satisfaction was also explored. This study is conducted in a real learning context. Although experimental research is the only way to draw a definite causal conclusion, investigating in a real learning context will help to gain a preliminary understanding of the possible causal processes (Obergriesser and Stoeger, 2020).

### Conclusion

The results offered partial support for the models we constructed. The conclusions emerging from the present study are as follows: First, students using different learning strategies could stimulate more positive emotions and less negative emotions. Second, positive emotions positively predicted learning satisfaction, while negative emotions negatively predicted learning satisfaction. Third, learning strategies had indirect effects on learning satisfaction through academic emotions. Lastly, behavioral engagement had a moderating effect on the relationship between learning strategies and satisfaction. The current research combined learning strategies with academic emotions, behavioral engagement, and social interaction to clarify how these variables work together to predict online learning satisfaction, which extends the influence of these variables on student's learning. Our findings suggested that learning strategies, academic emotions, and behavioral engagement are important variables influencing learning satisfaction. Therefore, this study is significant in that it provides empirical information on the importance of learning strategies, academic emotions, and behavioral engagement in online learning satisfaction, particularly by highlighting the mediating role of positive emotions and negative emotions.

### Mediating Effect of Academic Emotions

By constructing a mediating model, we found that metacognitive self-regulation and elaboration strategies had indirect effects on learning satisfaction through academic emotions, but the indirect effects of organization strategies were not significant (partially supporting H3). This finding showed that more metacognitive self-regulation and elaboration strategies were used to promote the positive and high arousal emotions of students to improve their learning satisfaction. However, in this study, the indirect effect of organization strategies on learning satisfaction was not significant. First, we examined that learning strategies can influence academic emotions. Metacognitive self-regulation and elaboration strategies could significantly positively predict positive emotions and negatively predict negative emotions, while organization strategies had no significant predictive effect (partially supporting H1). Theoretically, the influence of academic emotions and learning strategies could go in both directions (Pekrun, 2006; Pekrun and Perry, 2014). Thus, our findings support the CVT from which we drew this hypothesis. However, in direct contradiction to H1, organization strategies were unrelated to academic emotions. The relationship between organizational strategies and academic emotions is controversial. For example, King and Areepattamannil's (2014) findings showed that the use of organization strategies was positively associated with positive emotions but not correlated with negative emotions among secondary school students. Muis et al. (2015b) found in a study that the positive emotions of fifth-grade students were not related to how often they used organization strategies, but negative emotions were strongly correlated with organization strategies. The findings of these previous studies are all based on the influence of academic emotion on learning strategies, and there is still a lack of literature on the influence of learning strategies on academic emotion. One explanation for these findings may be that students are asked to complete tasks that fully demonstrate their learning abilities. However, these tasks are not important or necessary for students to use deep organization strategies to accomplish them (Obergriesser and Stoeger, 2020). Therefore, the organization strategies in this study are not enough to influence their academic emotions. Second, we found that positive emotions significantly positively predicted learning satisfaction, while negative emotions significantly negatively predicted learning satisfaction (supporting H2). This result was consistent with those of previous studies that academic emotions were important predictors of learning satisfaction and academic performance (Artino and Jones, 2012; Lee et al., 2021) and verified that academic emotions influenced learning achievement in the CVT (Pekrun, 2006). Specifically, Lee et al. (2021) indicated that the more positive emotions nursing graduate students had, the higher their learning satisfaction, highlighting the important role of academic emotions in graduate study. Pekrun et al. (2002) also proposed the influence of academic emotions on students' cognitive processes and academic performance, which is related to mental health (e.g., learning satisfaction). Lastly, we also found that elaboration and organization strategies significantly positively predicted learning satisfaction, which was consistent with some study results of Choi (2016) and Kasalak and Dagyar (2020). These conclusions verified that learning strategies had a positive influence on the learning achievement in the CVT (Pekrun, 2006). However, metacognitive self-regulation strategies could not significantly predict learning satisfaction. This phenomenon is possibly due to the fact that metacognitive self-regulation strategies belonged to deep-level learning strategies, and their use imposed higher requirements on the student's metacognitive abilities (Obergriesser and Stoeger, 2020). Thus, the relationship between metacognitive self-regulation strategies and learning satisfaction could be not a simple linear correlation. Although online students have more choices and opportunities to use learning strategies freely, they often report using them frequently but are not able to use them competently (Obergriesser and Stoeger, 2015). This phenomenon indicates that the students may give up their current learning strategies and adopt other more effective learning strategies if they have poor academic performance when using metacognitive self-regulation strategies.

### Moderating Effect of Behavioral Engagement and Social Interaction

After constructing two moderating models, we found that behavioral engagement had a significant moderating effect on the relationship between learning strategies and satisfaction, but the moderating effect of social interaction was not significant (partially supporting H4).

For students who use low learning strategies, high behavioral engagement enhanced their learning satisfaction. By contrast, students with high learning strategies, high behavioral engagement weakened their learning satisfaction. This finding shows that although behavioral engagement in online learning could promote learning satisfaction, its promotion effect largely depended on the level of use of learning strategies. When students used fewer learning strategies, their learning initiative was not high, and high behavioral engagement would encourage them to study more actively, thus leading to higher learning satisfaction (Gray and DiLoreto, 2016; Luo et al., 2019). However, when students used more learning strategies, the higher behavioral engagement would reduce learning satisfaction. This phenomenon is probably because the student's learning process involved strong planning and was targeted, and high engagement increased the external cognitive load (Gong et al., 2018). The high external cognitive load would influence the learning process, resulting in low learning satisfaction (Hawlitschek and Joeckel, 2017). However, for social interaction, we did not find a moderating effect. As mentioned in the literature review, danmakus related to learning content may improve learning satisfaction, while unrelated danmakus may reduce learning satisfaction (Zhang et al., 2019). This phenomenon is possibly due to the fact that many students send a lot of danmakus irrelevant to the learning content, which interfered with the learning process. In addition, most students did not send danmakus or rarely send them. Hence, these findings partially supported H4.

### Educational Implications

First, monitoring their learning process and making effective learning decisions are difficult for students, resulting in great differences in autonomous learning efficiency among individuals (Dunlosky and Rawson, 2012). Therefore, from the perspective of online instructional design, teachers should pay attention to guiding and helping students to use more effective and deep learning strategies, such as metacognitive self-regulation and elaboration strategies. Students need to use these strategies accurately to monitor and regulate learning behavior to optimize the learning process and results. The students use metacognitive self-regulation strategies (i.e., planning, monitoring, and regulation) to control their cognition and behavior, while they use elaboration strategies to connect new knowledge with existing knowledge in their brain (Obergriesser and Stoeger, 2020). This behavior was more likely to achieve better academic performance. Before a class, teachers can design relevant questionnaires to analyze the characteristics of students and their learning to help the students choose appropriate learning strategies based on their situation and previous experience. In addition, the course of “strategic learning” can be formulated to make the students clearly understand the advantages and disadvantages of their learning strategies use. This activity may promote the students to better manage the learning process and increase their positive emotions.

Second, from the perspective of online learning platform development, learning analysis technology based on artificial intelligence and big data can be introduced. For example, an online teaching feedback mechanism can be introduced to monitor and feedback the student's use of learning strategies and actively give adjustment suggestions to the students. Especially at the beginning of learning, the students will have positive emotions, such as curiosity and enjoyment, due to the freshness of online learning. However, the intensity and frequency of these positive emotions gradually decrease as the learning progresses. Previous studies have shown that positive emotions can promote students to seek actively learning opportunities and resources and participate more actively in learning activities (Zhen et al., 2017). Therefore, teachers should especially pay attention to guiding and helping students to conduct emotional self-regulation to improve effectively the learning satisfaction and results. Online learning platforms can also introduce technologies, such as affective computing, to develop affective teaching agents to acquire, evaluate, and give feedback on emotions and provide effective support to encourage students to produce positive emotions.

Finally, behavioral engagement, as a core variable to improve the quality of online learning, can help teachers clarify the online learning activity design from the perspective of online instructional design. Enhancing the quality of online courses by intervening and optimizing the design of the online teaching activities to promote student's learning satisfaction (Caskurlu et al., 2021). From the perspective of online learning platform development, user portrait and visualization technology can be introduced to guide and help students who use different levels of learning strategies to make appropriate behavioral engagement. In addition, relevant data collection tools, such as functional magnetic resonance imaging (fMRI) and electroencephalogram (EEG), can be developed to obtain the student's behavioral data and provide teachers and students feedback (Abreu et al., 2018). Students that use low learning strategies should be encouraged to reflect on learning and guided to make more behavioral engagement, such as the introduction of excellent online learning resources, the construction of active learning activities, and atmosphere (Shi et al., 2021). Students who use high learning strategies should be guided and helped to reduce unnecessary behavioral engagement.

## Limitations and Directions for Future Research

Several limitations should be acknowledged in this study that can profitably be addressed to stimulate future research. First, the sample size of participants is limited. In future research, data can be collected from more participants from different schools and different age levels. Second, we only evaluated three academic emotions (i.e., enjoyment, boredom, and frustration). These three emotions are the strongest and most frequently experienced emotions in school. However, many other emotions are also related to learning (e.g., confusion, curiosity, and pride, etc.) that need further research. Third, in this study, we first verify whether learning strategies can influence academic emotions. In future research, we will do a six-month follow-up study, using T1 (learning strategies), T2 (academic emotions), and T3 (learning strategies) as three-time points, and conduct a cross-lagged regression to further disentangle possible causal effects. Forth, in this study, students' academic emotions were derived from their self-report. In the future, EEG, fMRI, eye-tracking technologies, and other facial expression analysis tools combined with a face reader technology can be considered to conduct an in-depth study on academic emotions and cognitive neural mechanisms based on a single academic emotion (e.g., anxiety when asking questions) or a single academic emotion function (e.g., learning interest). Therefore, in the future, more advanced technologies and tools combined with educational neuroscience, starting from the specific application of the online student's learning strategies and changes in academic emotions at each stage, should be used.

## Data Availability Statement

The raw data supporting the conclusions of this article will be made available by the authors, without undue reservation.

## Ethics Statement

Ethical review and approval was not required for the study on human participants in accordance with the local legislation and institutional requirements. The patients/participants provided their written informed consent to participate in this study.

## Author Contributions

CW contributed to the study conception and design. Material preparation and data collection were performed by CW and JL. Data analysis was performed by BJ. The first draft of the manuscript was written by BJ and CW and all authors commented on previous versions of the manuscript. All authors read and approved the final manuscript.

## Funding

This work was supported by Humanities and Social Sciences projects of the Ministry of Education [18YJA760020]; The Effect and mechanism of Network environment on College Students' Belief in Life [2016P015]; and Institute of Psychology, Chinese Academy of Sciences [GJ202011].

## Conflict of Interest

The authors declare that the research was conducted in the absence of any commercial or financial relationships that could be construed as a potential conflict of interest.

## Publisher's Note

All claims expressed in this article are solely those of the authors and do not necessarily represent those of their affiliated organizations, or those of the publisher, the editors and the reviewers. Any product that may be evaluated in this article, or claim that may be made by its manufacturer, is not guaranteed or endorsed by the publisher.

## References

[B1] Abreu R. Leal A. Figueiredo P. (2018). EEG-informed fMRI: a review of data analysis methods. Front. Hum. Neurosci. 29:29. 10.3389/fnhum.2018.00029 29467634 PMC5808233

[B2] Ahmed W. Greetje V. D. W. Kuyper H. Minnaert A. (2013). Emotions, self-regulated learning, and achievement in mathematics: a growth curve analysis. J. Educ. Psychol. 105, 150–161. 10.1037/a0030160

[B3] Alexander P. A. Graham S. Harris K. R. (1998). A perspective on strategy research: progress and prospects. Educ. Psychol. Rev. 10, 129–154. 10.1023/A:1022185502996

[B4] Al-Fraihat D. Joy M. Sinclair J. (2020). Evaluating E-learning systems success: an empirical study. Comput. Human Behav. 102, 67–86. 10.1016/j.chb.2019.08.004

[B5] Artino Jr A. R. Jones K. D. II. (2012). Exploring the complex relations between achievement emotions and self-regulated learning behaviors in online learning. Internet High. Educ. 15, 170–175. 10.1016/j.iheduc.2012.01.006

[B6] Baber H. (2020). Determinants of students' perceived learning outcome and satisfaction in online learning during the pandemic of COVID-19. J. Educ. E-Learn. Res. 7, 285–292. 10.20448/journal.509.2020.73.285.292

[B7] Caskurlu S. Richardson J. C. Alamri H. A. Chartier K. Farmer T. Janakiraman S.. (2021). Cognitive load and online course quality: insights from instructional designers in a higher education context. Br. J. Educ. Technol. 52, 584–605. 10.1111/bjet.13043

[B8] Chao C.-M. (2019). Factors determining the behavioral intention to use mobile learning: an application and extension of the UTAUT model. Front. Psychol. 10:1652. 10.3389/fpsyg.2019.01652 31379679 PMC6646805

[B9] Choi B. (2016). How people learn in an asynchronous online learning environment: the relationships between graduate students' learning strategies and learning satisfaction. Can. J. Learn. Technol. 42, 1–15. 10.21432/T24K7R

[B10] Dai H. M. Teo T. Rappa N. A. Huang F. (2020). Explaining Chinese university students' continuance learning intention in the MOOC setting: a modified expectation confirmation model perspective. Comput. Educ. 150:103850. 10.1016/j.compedu.2020.103850

[B11] de la Fuente J. Peralta-Sánchez F. J. Martínez-Vicente J. M. Santos F. H. Fadda S. Gaeta-González M. L. (2020). Do learning approaches set the stage for emotional well-being in college students? Sustainability 12:6984. 10.3390/su12176984

[B12] D'Mello S. Graesser A. (2012). Dynamics of affective states during complex learning. Learn. Instruct. 22, 145–157. 10.1016/j.learninstruc.2011.10.001

[B13] Duncan T. G. McKeachie W. J. (2005). The making of the motivated strategies for learning questionnaire. Educ. Psychol. 40, 117–128. 10.1207/s15326985ep4002_6

[B14] Dunlosky J. Rawson K. A. (2012). Overconfidence produces underachievement: inaccurate self evaluations undermine students' learning and retention. Learn. Instruct. 22, 271–280. 10.1016/j.learninstruc.2011.08.003

[B15] Fredricks J. A. Blumenfeld P. C. Paris A. H. (2004). School engagement: potential of the concept, state of the evidence. Rev. Educ. Res. 74, 59–109. 10.3102/00346543074001059

[B16] Gong C. Li Q. Gong Y. (2018). Issues on learning engagement in smart learning environment. e-Educ. Res. 39, 83–89. 10.13811/j.cnki.eer.2018.06.014

[B17] Gong S. Han Y. Wang L. Gao L. Xiong J. (2016). The relationships among task value, academic emotions and online learning satisfaction. e-Educ. Res. 37, 72–77. 10.13811/j.cnki.eer.2016.03.010

[B18] Gray J. A. DiLoreto M. (2016). The effects of student engagement, student satisfaction, and perceived learning in online learning environments. Int. J. Educ. Leadersh. Prep. 11. Retrieved from: https://eric.ed.gov/?id=EJ1103654 (Accessed May, 2016).

[B19] Guo X. Wu F. Zheng X. (2019). “What motives learner to learn in MOOC? An investigation of Chinese University MOOC*,”* in *Paper Presented at the 2019 International Joint Conference on Information, Media and Engineering (IJCIME)* (Osaka).

[B20] Hawlitschek A. Joeckel S. (2017). Increasing the effectiveness of digital educational games: the effects of a learning instruction on students' learning, motivation and cognitive load. Comput. Hum. Behav. 72, 79–86. 10.1016/j.chb.2017.01.040

[B21] Hew K. F. Hu X. Qiao C. Tang Y. (2020). What predicts student satisfaction with MOOCs: a gradient boosting trees supervised machine learning and sentiment analysis approach. Comput. Educ. 145:103724. 10.1016/j.compedu.2019.103724

[B22] Hodges C. Moore S. Lockee B. (2020). The difference between emergency remote teaching and online learning. Educ. Rev. 27, 1–12. Retrieved from: https://er.educause.edu/articles/2020/3/the-difference-between-emergency-remote-teaching-and-online-learning (accessed June 15, 2020).

[B23] Jiang H. Islam A. A. Gu X. Spector J. M. (2021). Online learning satisfaction in higher education during the COVID-19 pandemic: a regional comparison between Eastern and Western Chinese universities. Educ. Inform. Technol. 10.1007/s10639-021-10519-x. [Epub ahead of print]. 33814959 PMC8010491

[B24] Jung I. Choi S. Lim C. Leem J. (2002). Effects of different types of interaction on learning achievement, satisfaction and participation in web-based instruction. Innov. Educ. Teach. Int. 39, 153–162. 10.1080/14703290252934603

[B25] Karakaya K. (2021). Design considerations in emergency remote teaching during the COVID-19 pandemic: a human-centered approach. Educ. Technol. Res. Dev. 69, 295–299. 10.1007/s11423-020-09884-0 33250609 PMC7679059

[B26] Kasalak G. Dagyar M. (2020). University student satisfaction, resource management and metacognitive learning strategies. Teach. Curric. 20, 73–85. 10.15663/tandc.v20i1.343

[B27] Ke F. Kwak D. (2013). Constructs of student-centered online learning on learning satisfaction of a diverse online student body: a structural equation modeling approach. J. Educ. Comput. Res. 48, 97–122. 10.2190/EC.48.1.e

[B28] King R. B. Areepattamannil S. (2014). What students feel in school influences the strategies they use for learning: academic emotions and cognitive/meta-cognitive strategies. J. Pac. Rim Psychol. 8, 18–27. 10.1017/prp.2014.3

[B29] Kong B. Lu H. (2012). Compilation of questionnaire on self-regulated learning strategies for middle school students. J. Sichuan Norm. Univ. 39, 129–134. 10.13734/j.cnki.1000-5315.2012.05.027

[B30] Lee M. Na H. M. Kim B. Kim S. Y. Park J. Choi J. Y. (2021). Mediating effects of achievement emotions between peer support and learning satisfaction in graduate nursing students. Nurse Educ. Pract. 52:103003. 10.1016/j.nepr.2021.103003 33774568

[B31] Leng J. Zhu J. Wang X. Gu X. (2016). Identifying the potential of Danmaku video from Eye Gaze Data, in Paper Presented at the 2016 IEEE 16th International Conference on Advanced Learning Technologies (ICALT) (Austin, TX).

[B32] Luo Y. Xie M. Lian Z. (2019). Emotional engagement and student satisfaction: a study of Chinese college students based on a nationally representative sample. Asia Pac. Educ. Res. 28, 283–292. 10.1007/s40299-019-00437-5

[B33] Mariza C. Irini D. Anastasia E. Aggeliki L. (2015). Motivational and affective determinants of self-regulatory strategy use in elementary school mathematics. Educ. Psychol. 35, 835–850. 10.1080/01443410.2013.822960

[B34] Méndez-Aguado C. Aguilar-Parra J. M. Álvarez J. F. Trigueros R. Fernández-Archilla J. A. (2020). The influence of emotions, motivation and habits in the academic performance of primary education students in French as a foreign language. Sustainability 12:2531. 10.3390/su12062531

[B35] Moore J. C. (2005). The Sloan Consortium Quality Framework and the Five Pillars. The Sloan Consortium (Newburyport, MA).

[B36] Moore M. G.. (1989). Three types of interaction. Am. J. Distance Educ. 3, 1–6. 10.1080/08923648909526659

[B37] Muilenburg L. Y. Berge Z. L. (2005). Student barriers to online learning: a factor analytic study. Dist. Educ. 26, 29–48. 10.1080/01587910500081269

[B38] Muis K. R. Pekrun R. Sinatra G. M. Azevedo R. Trevors G. Meier E.. (2015a). The curious case of climate change: testing a theoretical model of epistemic beliefs, epistemic emotions, and complex learning. Learn. Instruct. 39, 168–183. 10.1016/j.learninstruc.2015.06.003

[B39] Muis K. R. Psaradellis C. Lajoie S. P. Di Leo I. Chevrier M. (2015b). The role of epistemic emotions in mathematics problem solving. Contemp. Educ. Psychol. 42, 172–185. 10.1016/j.cedpsych.2015.06.003

[B40] Murayama K. Pekrun R. Lichtenfeld S. Vom Hofe R. (2013). Predicting long-term growth in students' mathematics achievement: the unique contributions of motivation and cognitive strategies. Child Dev. 84, 1475–1490. 10.1111/cdev.12036 23278807

[B41] Nagy J. T. (2018). Evaluation of online video usage and learning satisfaction: an extension of the technology acceptance model. Int. Rev. Res. Open Distrib. Learn. 19, 159–185. 10.19173/irrodl.v19i1.2886

[B42] Noteborn G. Carbonell K. B. Dailey-Hebert A. Gijselaers W. (2012). The role of emotions and task significance in virtual education. Internet High. Educ. 15, 176–183. 10.1016/j.iheduc.2012.03.002

[B43] Obergriesser S. Stoeger H. (2015). The role of emotions, motivation, and learning behavior in underachievement and results of an intervention. High Abil. Stud. 26, 167–190. 10.1080/13598139.2015.1043003

[B44] Obergriesser S. Stoeger H. (2020). Students' emotions of enjoyment and boredom and their use of cognitive learning strategies–How do they affect one another? Learn. Instruct. 66:101285. 10.1016/j.learninstruc.2019.101285

[B45] Pekrun R. (2006). The control-value theory of achievement emotions: assumptions, corollaries, and implications for educational research and practice. Educ. Psychol. Rev. 18, 315–341. 10.1007/s10648-006-9029-9

[B46] Pekrun R. Elliot A. J. Maier M. A. (2009). Achievement goals and achievement emotions: testing a model of their joint relations with academic performance. J. Educ. Psychol. 101, 115–135. 10.1037/a0013383

[B47] Pekrun R. Goetz T. Frenzel A. C. Barchfeld P. Perry R. P. (2011). Measuring emotions in students' learning and performance: the Achievement Emotions Questionnaire (AEQ). Contemp. Educ. Psychol. 36, 36–48. 10.1016/j.cedpsych.2010.10.002

[B48] Pekrun R. Goetz T. Titz W. Perry R. P. (2002). Academic emotions in students' self-regulated learning and achievement: a program of qualitative and quantitative research. Educ. Psychol. 37, 91–105. 10.1207/S15326985EP3702_4

[B49] Pekrun R. Perry R. P. (2014). Control-value theory of achievement emotions, in International Handbook of Emotions in Education, eds Pekrun R. Linnenbrink-Garcia L. (https://www.google.com/search?sxsrf=AOaemvK2Som0JMRwYfBFZ2jbBL1tz8ry7g:1630601351743&q=London&stick=H4sIAAAAAAAAAOPgE-LUz9U3ME4xzStW4gAxTbIKcrRUs5Ot9 London: Routledge), 130–151.

[B50] Pintrich P. R. De Groot E. V. (1990). Motivational and self-regulated learning components of classroom academic performance. J. Educ. Psychol. 82, 33–40. 10.1037/0022-0663.82.1.33

[B51] Rabin E. Kalman Y. M. Kalz M. (2019). An empirical investigation of the antecedents of learner-centered outcome measures in MOOCs. Int. J. Educ. Technol. High. Educ. 16, 1–20. 10.1186/s41239-019-0144-3

[B52] Salam M. Farooq M. S. (2020). Does sociability quality of web-based collaborative learning information system influence students' satisfaction and system usage? Int. J. Educ. Technol. High. Educ. 17, 1–39. 10.1186/s41239-020-00189-z

[B53] Sewart D. (1993). Student support systems in distance education. Open Learn. J. Open Distance e-Learn. 8, 3–12. 10.1080/0268051930080302

[B54] Shi Y. Tong M. Long T. (2021). Investigating relationships among blended synchronous learning environments, students' motivation, and cognitive engagement: a mixed methods study. Comput. Educ. 168:104193. 10.1016/j.compedu.2021.104193

[B55] Shrout P. E. Bolger N. (2002). Mediation in experimental and nonexperimental studies: new procedures and recommendations. Psychol. Methods 7, 422–445. 10.1037/1082-989X.7.4.422 12530702

[B56] Wolters C. A. Pintrich P. R. Karabenick S. A. (2005). Assessing academic self-regulated learning, in What Do Children Need to Flourish? eds Moore K. A. Lippman L. H. (https://www.google.com/search?sxsrf=AOaemvIkZGt6zItpTa1tvvKrmPqgIk4IA:1630601612724&q=Berlin&stick=H4sIAAAAAAAAAOPgE-LUz9U3MImvKjFQ4gAxDU3NC Berlin: Springer), 251–270.

[B57] Wu Q. Sang Y. Huang Y. (2019). Danmaku: a new paradigm of social interaction via online videos. ACM Trans. Soc. Comput. 2, 1–24. 10.1145/3329485

[B58] Xiong J. Gong S. Frenzel A. C. (2012). The relationship between mathematics academic emotions, learning strategies and mathematics achievements. Educ. Res. Exp. 38, 89–92.

[B59] Yang J. (2014). The effect of the instructor on learning process and effectiveness in online video courses (doctoral thesis). Central China Normal University, Wuhan, China. Available online at: https://kns.cnki.net/KCMS/detail/detail.aspx?dbname=CDFDLAST2015andfilename=1015520788.nh

[B60] Yang J. Wu C. Pi Z. Xie H. (2019). Facilitating learning or interfering learning: a meta-analysis of impact of danmaku on learning. e-Educ. Res. 40, 84–90. 10.13811/j.cnki.eer.2019.06.011

[B61] Zhang Y. Qian A. Pi Z. Yang J. (2019). Danmaku related to video content facilitates learning. J. Educ. Technol. Syst. 47, 359–372. 10.1177/0047239518811933

[B62] Zhen R. Liu R.-D. Ding Y. Wang J. Liu Y. Xu L. (2017). The mediating roles of academic self-efficacy and academic emotions in the relation between basic psychological needs satisfaction and learning engagement among Chinese adolescent students. Learn. Individ. Differ. 54, 210–216. 10.1016/j.lindif.2017.01.017

