# Supplementary material for: Student's Learning Strategies and Academic Emotions: Their Influence on Learning Satisfaction During the COVID-19 Pandemic
Source: Front Psychol. 2021 Sep 24;12:717683. doi: 10.3389/fpsyg.2021.717683 (PMC8500055; doi:10.3389/fpsyg.2021.717683)
Supplement: Supplementary file 1 [file Data_Sheet_1.docx]

**Appendix A: Learning strategy Scales**

**Elaboration**

53. When I study for this online course, I pull together information from different sources, such as lectures, readings, and discussions.

62. I try to relate ideas in this online course to those in other courses whenever possible.

64. When reading for this online course, I try to relate the material to what I already know.

67. When I study for this online course, I write brief summaries of the main ideas from the readings and my class notes.

69. I try to understand the material in this online course by making connections between the readings and the concepts from the lectures.

81. I try to apply ideas from this online course readings in other class activities such as lecture and discussion.

**Organization**

32. When I study the readings for this online course, I outline the material to help me organize my thoughts.

42. When I study for this online course, I go through the readings and my class notes and try to find the most important ideas.

49. I make simple charts, diagrams, or tables to help me organize online course material.

63. When I study for this online course, I go over my class notes and make an outline of important concepts.

**Metacognitive self-regulation**

33. During this online course time I often miss important points because I’m thinking of other things. (REVERSED)

36. When reading for this online course, I make up questions to help focus my reading.

41. When I become confused about something I’m reading for this online course, I go back and try to figure it out.

44. If the online course readings are difficult to understand, I change the way I read the material.

54. Before I study new online course material thoroughly, I often skim it to see how it is organized.

55. I ask myself questions to make sure I understand the material I have been studying in this online course.

56. I try to change the way I study in order to fit the online course requirements and the instructor’s teaching style.

57. I often find that I have been reading for this online course but don’t know what it was all about. (REVERSED)

61. I try to think through a topic and decide what I am supposed to learn from it rather than just reading it over when studying for this online course.

76. When studying for this online course I try to determine which concepts I don’t understand well.

78. When I study for this online course, I set goals for myself in order to direct my activities in each study period.

79. If I get confused taking notes in online course, I make sure I sort it out afterwards.

**Appendix B: Academic Emotion Scales**

**Enjoyment**

1 I am enjoying the online course.

2 I am very excited when studying the online course.

3 I am very happy to understand the learning material of the online course.

4 I am very happy to study in the online course, it makes me want to learn more related content.

**Frustration**

5 I felt very frustrated when studying the online course.

6 When studying the online course, I was very irritated.

7 When studying the online course, I felt like I was wasting time.

8 When studying the online course, I was very angry.

**Boredom**

9 I feel bored while studying the online course.

10 I think the online course is very boring.

11 I find my mind wandering while I studying the online course.

12 I don't like the learning material of the online course.

13 The online course is so boring that I find myself daydreaming.

**Appendix C: Learning Satisfaction Scales**

**Teacher instruction**

1 Overall, I am satisfied with the teaching of this online course.

2 Overall, I am satisfied with the teacher's explanation in the online course.

3 The tips of the online course help me study.

4 Overall, I am satisfied with the content of the online course.

5 The learning content in the online course attracted me and helped me.

6 The tasks in the online course are challenging.

**Learning content**

7 The task volume and task difficulty in this online course are moderate.

8 Overall, I am satisfied with the learning environment and equipment for the online course.

9 I am satisfied with the teaching format of the online course.

10 I am satisfied with the normal operation of the teaching of the online course.
